# Impact of XPF rs2276466 polymorphism on cancer susceptibility: a meta-analysis

**DOI:** 10.1042/BSR20181785

**Published:** 2019-05-24

**Authors:** Yezhou Liu, Kun Liu, Xueru Zhao, Yidan Sun, Ning Ma, Longmei Tang, Haitao Yang, Xia Gao, Lina Yan, Meina Yuan, Bingshuang Wang, Xiaolin Zhang, Jinhai Jia

**Affiliations:** 1 Academy of Basic Medicine, Hebei Medical University, Shi Jiazhuang, China; 2 Department of Joint and Trauma Orthopedics, Bayingolin People’s Hospital, Korla, Xinjiang, China; 3 Department of Social Medicine and Health Care Management, School of Public Health, Hebei Medical University, Shi Jiazhuang, China; 4 Department of Epidemiology and Statistics, School of Public Health, Hebei Medical University, Hebei Province Key Laboratory of Environment and Human Health, Shi Jiazhuang, China; 5 Outpatient Clinics, Hebei Medical University, Shi Jiazhuang, China

**Keywords:** cancer, meta-analysis, polymorphism, XPF

## Abstract

Association between the xeroderma pigmentosum complementation group F (XPF)rs2276466 located in the excision repair cross complementation group 4 (ERCC4) gene and cancer susceptibility has been widely investigated. However, results thus far have remained controversial. A meta-analysis was performed to identify the impact of this polymorphism on cancer susceptibility. PubMed, Embase and Science-Web databases were searched systematically up to May 20, 2018, to obtain all the records evaluating the association between the rs2276466 polymorphism and the risk of all types of cancers. We used the odds ratio (OR) as a measure of effect, and pooled the data in a Mantel-Haenszel weighed random-effects meta-analysis to provide a summary estimate of the impact of this polymorphism on gastrointestinal cancer, neurogenic cancer and other cancers (breast cancer and SCCHN). All the analyses were carried out in STATA 14.1.11 case–control studies that consisted of 5730 cases and 6756 controls, were eventually included in our meta-analysis. The significant association was observed between the XPFrs2276466 polymorphism and neurogenic cancer susceptibility (recessive model: OR = 1.648, 95% CI = 1.294–2.098, *P*<0.001). Furthermore, no significant impact of this polymorphism was detected on decreased gastrointestinal cancer risk (dominant model: OR = 1.064, 95%CI = 0.961–1.177, *P* = 0.233). The rs2276466 polymorphism might play different roles in carcinogenesis of various cancer types. Current evidence did not suggest that this polymorphism was directly associated with gastrointestinal susceptibility. However, this polymorphism might contribute to increased neurogenic cancer risk. More preclinical and epidemiological studies are still imperative for further evaluation

## Introduction

**Figure 1:**
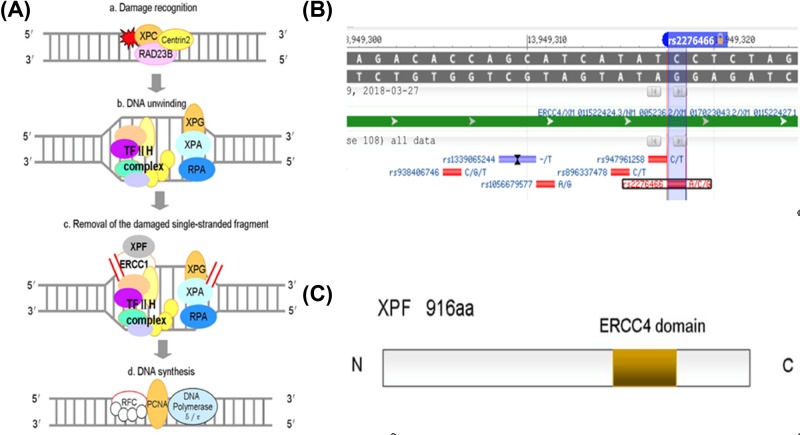
Structural characteristics and function of XPF as modified from KEGG and GeneBank database (**A**) NER involves at least four steps: (a) damage recognition by a complex of bound proteins including XPC, (b) unwinding of the DNA by the TFIIH complex that includes XPD, (c) removal of the damaged single-stranded fragment by molecules including an ERCC1/XPF complex and (d) synthesis by DNA polymerases. (**B**) The XPF gene map labeled with rs2276466, and four polymorphisms that have been commonly studied for their associations with cancer risk. (**C**) The XPF protein consists of 916 amino acids, containing an ERCC4 domain. Abbreviations: KEGG, Kyoto Encyclopedia of Genes and Genomes; NER, nucleotide excision repaired.

Nucleotide excision repair (NER) is the most versatile, well studied DNA repair mechanism in humans [1], mainly responsible for repairing bulky DNA damage, such as DNA adducts caused by UV radiation, mutagenic chemicals or chemotherapeutic drugs. The repair process includes excising and removing damaged nucleotides [2] and synthesizing to fill the resultant gap by using the complementary DNA strand as a template. Therefore, reduced DNA repair capacity (DRC) [3] may lead to genomic instability and carcinogenesis, and genes involved in the NER pathway are candidate cancer susceptibility genes. NER involves at least four steps (Figure 1A): (1) damage recognition by a complex of bound proteins including XPC; (2) unwinding of the DNA by the TFIIH complex that includes XPD; (3) removal of the damaged single-stranded fragment by molecules including an ERCC1/XPF complex; and (4) synthesis by DNA polymerases [4].

One of the NER genes, xeroderma pigmentosum complementation group F (XPF), also called excision repair cross-complimentary group 4 (ERCC4) [5], is located on chromosome 16p13.12, contains rs2276466 (Figure 1B). The XPF protein consists of 916 amino acids [6], containing an ERCC4 domain (Figure 1C) that is one of the nuclease families, in which essential meiotic endonuclease 1 (EME1) acts as an essential component of a Holliday junction resolvase to interact with MUS81 [7]. The ERCC4 domain is also necessary for forming a tight complex with ERCC1 as a structure-specific DNA repair endonuclease responsible for the 59-primer incision during DNA excision repair (Figure 1C). In addition to NER, this complex is suggested to play a role in removal of DNA inter-strand cross-links (ICL) [8] and DNA double-strand breaks (DSB) [9].

To date, a number of molecular epidemiological studies have been done to evaluate the association between XPFrs2276466 polymorphisms and different types of cancer risk in diverse populations. However, the results were inconsistent or even contradictory, partially because of the possible small effect of the polymorphism on cancer risked the relatively small sample size in each of published study. In addition, two recent meta-analyses have studied the association between XPF and risk of cancer [10]. However, that article and many published studies rarely referred to rs2276466. Therefore, we performed a comprehensive meta-analysis by including the most recent and relevant articles to identify statistical evidence of the association between XPFrs2276466 polymorphisms and risk of all cancers that have been investigated. Meta-analysis is an outstanding tool for summarizing the different studies. Not only can it overcome the problem of small size and inadequate statistical power of genetic studies of complex traits, but can also provide more reliable results than a single case–control study.

## Methods

### Literature search strategy

We first used three: electronic databases (EMBASE, PUBMED, Cochrane library databases and SCIENCE-WEB) to identify all case–control studies published to date on an association between rs2276466 polymorphisms and cancer risk (the last search update on May 20, 2018, using the search terms: “XPF/ERCC4”; “rs2276466’’; “cancer”; “sarcoma”; “neoplasia”; “malignancy”; “carcinoma”; “polymorphism” or “variant”). To expand the coverage of our searches, we further searched Chinese National Knowledge Infrastructure (CNKI) database (http://www.cnki.net/) (1979–) and Wanfang database, using the terms ‘‘rs2276466”; ’‘cancer” in Chinese. Additional published studies on this topic in the references of each publication were also hand reviewed. We further contacted study investigators to identify some unpublished or submitted studies. Only studies with a full-text article were included. The authors of published papers were also contacted directly, if crucial data were not reported in original papers. When more than one of the same patient populations were included in different publications, only the most recent or complete study with the largest sample size was included in this meta-analysis [11].

### Inclusion and exclusion criteria

Studies consistent with the following criteria were included in our meta-analysis: (1) assessing the association between the ERCC4/XPF rs2276466 and risk for cancer12; (2) case-control studies; (3) sufficient data (a detailed number of genotypes including C/C, C/G and G/G in both the case and the control group13); (4) English articles. Exclusion criteria were as follows:

1. Case only studies or case reports;
2. Meta-analyses or reviews;
3. Studies without detailed genotyping data;
4. Duplicate publications;
5. Low quality studies evaluated by the Newcastle-Ottawa Scale (NOS).

### Data extraction

Information was carefully extracted from all eligible studies by two independent investigators according to the inclusion criteria listed above. The following data were collected from each study: first author’s name, year of publication, ethnicity, type of cancer, source of controls and numbers of cases–controls in the XPF rs2276466 genotypes whenever possible. Meanwhile, studies investigating more than one kind of cancer were counted as individual data sets only in subgroup analyses by cancer type. We did not define any minimum number of patients to include in this meta-analysis. In case of articles reporting different ethnic groups and different countries or locations, we considered them different study samples for each category cited above.

### Statistical analysis

Odds ratios (ORs) and corresponding 95% confidence intervals (CIs) were used as the measure of effect to evaluate the strength of association between the XPF/rs2276466 polymorphism and cancer susceptibility. Data were pooled using the Mantel-Haenszel method (*P*<0.05 was considered as statistically significant). For this polymorphism, the dominant model (Dom.: C/C + C/G vs. G/G), recessive model (Rec.: C/C vs. C/G +G/G) were chosen to calculate the pooled ORs.

In view of the potential heterogeneity among studies with different cancer types, the random effects model (the Der-Simonian and Laird method [12]) was adopted. Heterogeneity among eligible studies was assessed by Cochran’s *Q* test. The *P*-value < 0.1 indicated significant heterogeneity according to the previous study [13]. We conducted stratified analyses by variables such as cancer types, and ethnicity. Notably, if significant heterogeneity was observed after stratified analysis, a meta-regression analysis was performed to explore the potential origin of heterogeneity. By sequentially excluding every single study, we conducted sensitivity analysis to identify stability of the results and check whether any single study contributed to the heterogeneity significantly [14].

Considering that enough studies should be included in order to observe the variation trend of the ORs effectively, we performed cumulative meta-analysis as evidence accumulated by time when 10 or more studies were included. We checked the symmetry of Begg’s funnel plot and the results of Begg’s test to assess the publication bias [15]. All of the statistical analyses were conducted by STATA (version 14.1; StataCorp, College Station, Tex)

## Results

### Identification and characteristics of eligible studies

**Figure 2:**
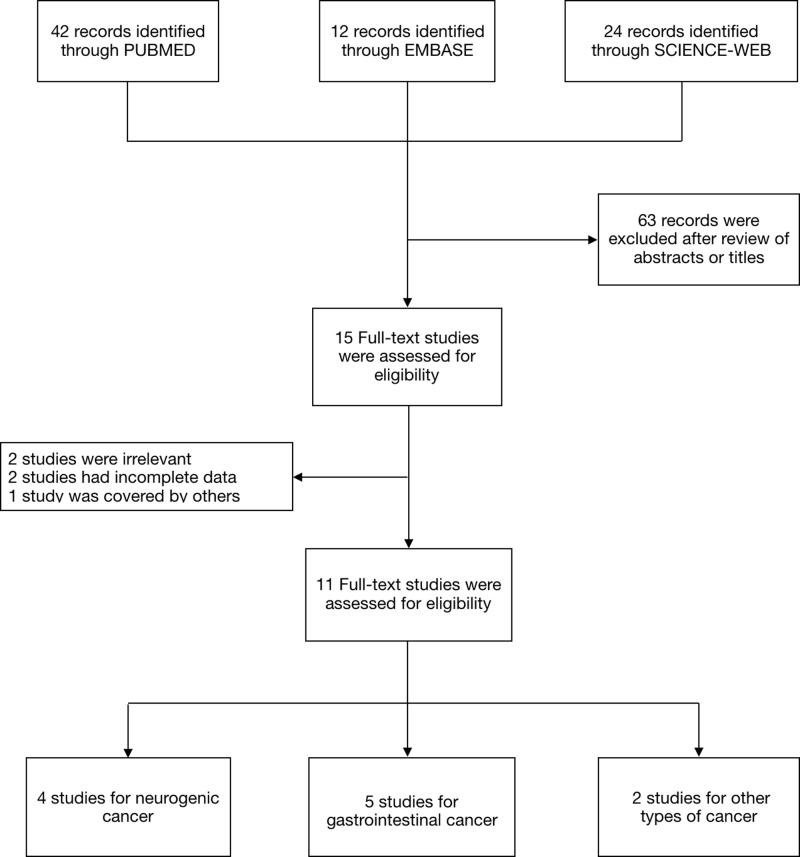
PRISMA flow diagram for study selection

After an initial search with duplicates discarded, a total of 78 records of publications were yielded. Following the predefined inclusion and exclusion criteria, eventually 11 articles with 11 case–control studies were included in this meta-analysis (details in Figure 2).

The basic characteristics of eligible studies were listed in Table 1 [10,16,17,18–25]. Among these eligible studies, there were four case–control studies that investigated association between the XPF/ERCC4 rs2276466 polymorphism and neurogenic cancer risk, and five articles focused on gastrointestinal cancer. A wide range of cancer types were covered by studies including glioma, neuroblastoma, SCCHN, gastric, colorectal and breast cancer. Regarding ethnicity, all of the eligible studies included solely Chinese subjects. As shown in Table 1, and we also summarized genotypes for all genetic models.

**Table 1:** Genotype distribution of rs2276476 polymorphism

First author	Year	Cancer Type	Ethnicity	Simple size		Quality scores
				Case	Control	
Zhou [23]	2014	Glioma	Chinese	225	262	13
Wang [21]	2013	Glioma	Chinese	330	652	12
Zhou [25]	2018	Neuroblastoma	Chinese	387	812	9
Cheng [20]	2013	Glioma	Chinese	207	236	10
Yu [19]	2012	SCCHN	Chinese	1040	1046	8
He [24]	2018	Gastric	Chinese	1141	1173	13
He [18]	2012	Gastric	Chinese	1125	1196	11
Zhang [22]	2013	Gastric	Chinese	331	355	11
Yang [10]	2015	Colorectal	Chinese	279	316	10
Hou [16]	2014	Colorectal	Chinese	204	204	12
Yang [17]	2013	Breast	Chinese	416	504	9

### Quantitative analysis

The eligible case–control studies were mainly composed of the following types of cancers (*N*=11): neurogenic cancer (*N*=4), gastrointestinal cancer (*N*=5) and others (*N*=2). Τhis brought about potential bias to the combined analysis. Additionally, the considerable inherent heterogeneity among different cancer types and significant statistical heterogeneity we observed (*P*_h_ = 0.001) indicated that it might not be informative to pool the data of all types of cancer into a single analysis. Therefore, we performed meta-analyses in the different cancer types, and the results are summarized in Table 2.

**Table 2:** Stratified analysis of rs2276466 polymorphism on cancer susceptibility

Variables	*N*	Case/Control	Dominant model		Recessive model		Additive model	
			OR	*P/P*_h_	OR	*P/P*_h_	OR	*P/P*_h_
Neurogenic	4	1149/1962	1.161	0.048/0.363	1.648	0.000/0.973	1.224	0.001/0.495
Gastrointestinal	5	3080/3224	1.064	0.233/0.359	1.017	0.876/0.811	1.046	0.294/0.362
Others	2	1501/1550	1.041	0.576/ 0.608	0.889	0.659/ 0.048	0.989	0.845/ 0.206
Total	11	5730/6756	1.080	0.036/0.525	1.174	0.122/0.017	1.072	0.019/0.105

### All types of cancer

**Figure 3:**
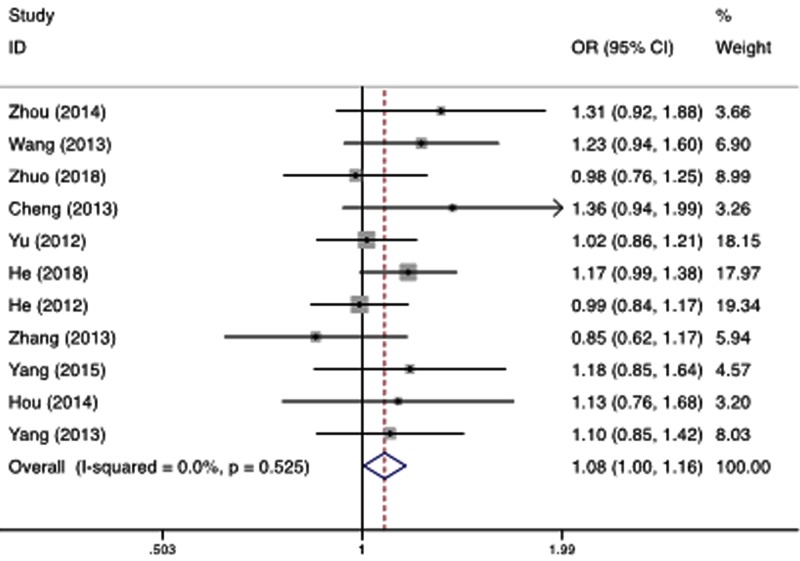
Forest plot for the association of the rs2276466 polymorphism with all types of cancer (dominant model: C/C + C/G vs. G/G)

A total of 5730 cases in all cancer types and 6756 controls were included between rs2276466 polymorphism and all types of cancer, which identified a significant association. We observed that variant allele carriers (C/C+C/G) had significantly higher risk for cancer under dominant (OR = 1.080, 95%CI = 1.005–1.161, *P*=0.036, Figure 3), recessive (OR = 1.174, 95%CI = 0.958–1.438, *P*=0.122) and additive model (OR = 1.072, 95%CI = 1.012–1.135, *P*=0.019). The heterogeneity was found in the recessive genetic model, then we did the stratified analysis in the different types.

### Neurogenic cancer

**Figure 4:**
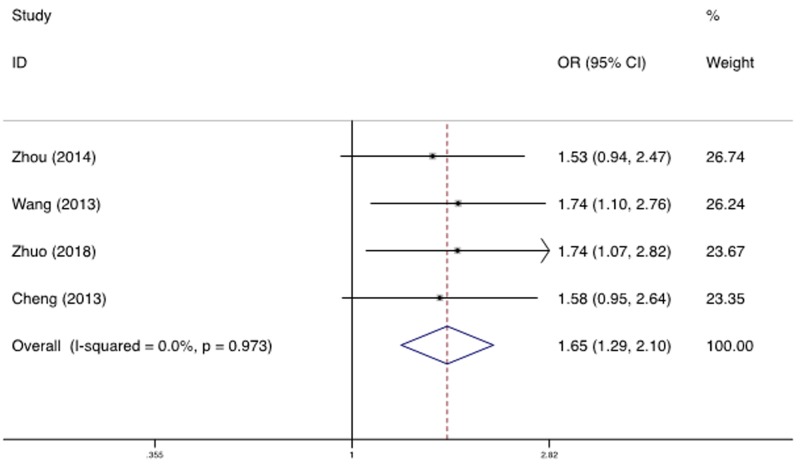
Forest plot for the association of the rs2276466 polymorphism with neurogenic cancer (recessive model: C/C vs. C/G+G/G)

A total of 1149 neurogenic cancer cases and 1962 controls were included between rs2276466 polymorphism and neurogenic cancer, which identified a significant association. We observed that variant allele carriers (C/C+C/G) had significantly higher risk for neurogenic cancer under dominant (OR = 1.161, 95%CI = 1.001–1.346, *P* = 0.048), recessive (OR = 1.648, 95%CI = 1.294–2.098, *P*<0.001, Figure 4) and additive model (OR = 1.224, 95%CI = 1.092–1.373, *P* = 0.001). No significant heterogeneity was found in any genetic model.

### Gastrointestinal cancer

A total of 3080 gastrointestinal cancer cases and 3224 controls were included in the meta-analysis. As shown in Table 2, no significant association was observed between rs2276466 polymorphism and gastrointestinal cancer risk under dominant (OR = 1.064, 95%CI = 0.961–1.177, *P*=0.233), recessive (OR = 1.017, 95%CI = 0.823–1.257, *P*=0.876) and additive model (OR = 1.046, 95%CI = 0.962–1.137, *P*=0.294). Cumulative meta-analysis obtained no significant association as evidence accumulated by time.

### Heterogeneity and sensitivity analyses

**Figure 5:**
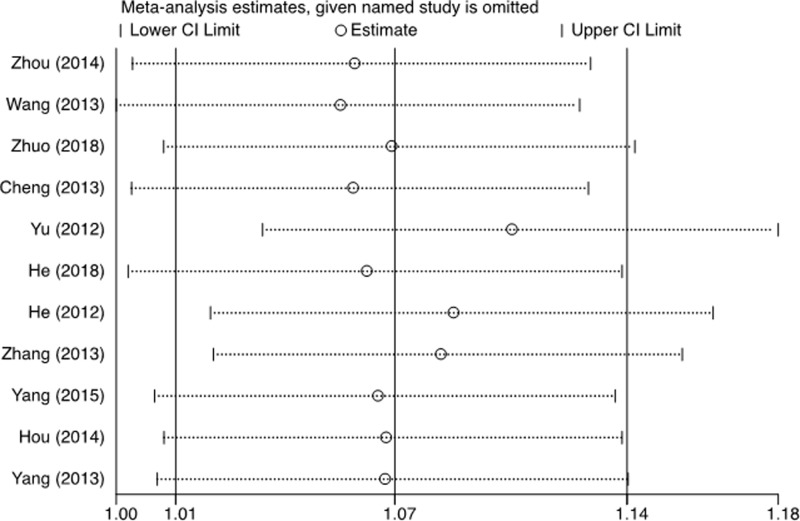
Sensitivity analysis on the association between the rs2276466 polymorphism and susceptibility of all cancers type (additive model: C. VS G.)

Substantial heterogeneities were observed among studies for the association between the XPF-rs2276466 polymorphism and all cancer risk in recessive model (*x*^2^ = 21.63, d*f* = 10, *P*=0.017). Therefore, we used the random-effects model that generated wider CIs. For the other two cancer types (i.e. neurogenic cancer and gastrointestinal cancer), no heterogeneity was found among studies or in stratification analyses in recessive models (*x*^2^ = 0.23, d*f* = 3, *P*=0.973; *x*^2^ = 1.59, d*f* = 4, *P*=0.811, respectively), and the fixed-effects model was performed [26]. The leave-one-out sensitivity analysis indicated that no single study changed the pooled ORs qualitatively (Figure 5).

### Publication bias

**Figure 6:**
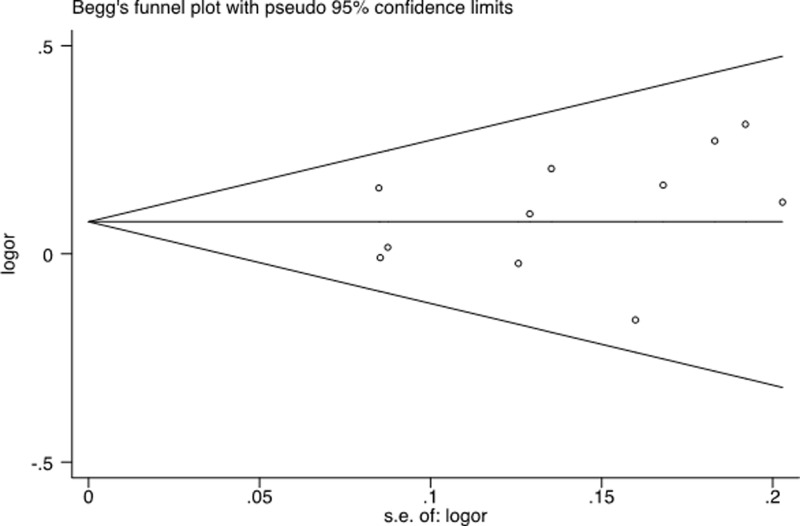
Begg’s funnel plot on publication bias for eligible studies that focused on the association of the rs2276466 polymorphism with all types of cancer susceptibility

The shapes of the funnel plots (Figure 6) seemed symmetrical, and Begg’s test suggested that there was no publication bias [27] for studies of XPF-rs2276466 associations with cancer risk in the current meta-analysis (recessive model: *P*=0.350; dominant model: *P*=0.213; additive model: *P*=0.119, no value, respectively). These findings indicated that bias from publications, if any, might not have a significant effect on the results of our meta-analysis for the association between the four commonly studied XPF polymorphisms and overall cancer risk.

## Discussion

As research has progressed, the genomic landscape of cancer has been gradually brought into light, and an increasing number of vital genes shared by various cancers [28,29], ERCC4/XPF for example, have been revealed in recent years. The XPF-rs2276466 polymorphism was widely reported to be associated with susceptibility of a wide range of cancers [30]. However, results remain conflicting in different types of cancer and a single study might be limited due to a relatively small sample size. Moreover, no conclusive study so far has reported a result that covered all available cancer types. Based on all published literature, we performed this meta-analysis to identify the association of the XPF-rs2276466 polymorphism with cancer susceptibility [31].

Our study, which derived an asymmetrical distribution of different cancer types, contained four studies for neurogenic cancer, five for gastrointestinal cancer and two for ‘others’ generating potential bias to the combined analysis of all cancer types. Moreover, considerable heterogeneity existed among different cancer types, which was confirmed by the significant statistical heterogeneity we obtained [32]. Current evidence indicated that the XPF-rs2276466 polymorphism might play various roles in different cancer types. Thus, it might be of little value to combine all data of different cancer types into a single analysis.

According to our meta-analysis of gastrointestinal cancer, we observed no significant association between this polymorphism and susceptibility of gastrointestinal cancer [33]. Cumulative meta-analysis suggested that no significant association was observed as evidence accumulated by time [34]. Theoretically, genetic variants in the ERCC4/XPF gene could change the regular function of this gene [35], disturb the DNA repair mechanism and increase cancer risk. Therefore, studies should pay more attention to the association between rs2276444 in the ERCC4 gene and gastrointestinal cancer.

To our knowledge, this is the most comprehensive meta-analysis that has investigated the association between the XPF-rs2276466 polymorphism and susceptibility of all available cancer types [22]. However, several limitations should be taken into consideration when explaining the results: (1) the number of studies included was relatively small for some cancer types, for example, only one study investigated breast cancer [17]; (2) due to insufficient information, stratified analysis could not be conducted by age, sex, treatment, drinking status, exposure to radiation and other factors [36]; (3) as only Chinese subjects were involved in the pooled analysis, the results might not be as relevant to other ethnicities [37].

In conclusion, our meta-analysis found that the impact of the XPF-rs2276466 polymorphism on the susceptibility of different cancers might be diverse. Current evidence did not suggest this polymorphism was directly associated with gastrointestinal cancer risk. However, we observed that the variant allele carriers might contribute to the risk for developing neurogenic cancer. Our results should be explained with some caution and be re-evaluated in the future when more studies with larger sample sizes are conducted.

## Abbreviations

CI: confidence interval
DRC: DNA repair capacity
ERCC4: excision repair cross complementation group 4
NER: nucleotide excision repair
OR: odds ratio
XPF: xeroderma pigmentosum complementation group F
